# Lectotypifications of six taxa in the Boraginales (Cordiaceae and Heliotropiaceae)

**DOI:** 10.3897/phytokeys.62.6259

**Published:** 2016-04-15

**Authors:** Christian Feuillet

**Affiliations:** 1 Department of Botany, MRC-166, National Museum of Natural History, Smithsonian Institution, P.O. Box 37012, Washington, DC 20013-7012, USA; 2 Department of Botany and Plant Pathology, 2082 Cordley Hall, Oregon State University, Corvallis OR 97331-2902, USA

**Keywords:** Cordia, Cordiaceae, Heliotropiaceae, lectotype, Myriopus, Tournefortia, Varronia

## Abstract

A large number of specimens used as original material for the description of new species were destroyed in the bombing of the Berlin-Dahlem herbarium, B, in 1943. Six lectotypes are designated here for *Cordia discolor* Cham., *Cordia multispicata* Cham., *Cordia tobagensis* Urb. and its variety *broadwayi* Urb. in the Cordiaceae and for *Tournefortia paniculata* Cham. and *Tournefortia ulei* Vaupel in the Heliotropiaceae.

## Introduction

In the last ten years, several lectotypes have been designated in the Cordiaceae and Heliotropiaceae (Miller in Cafferty and Jarvis 2004; Gottschling and Miller 2006; Miller 2007; Feuillet 2008, 2013; Stapf 2010; Stapf and Silva 2013). In the preparation for the treatment of the Boraginales, or Boraginaceae s.l., for the Flora of the Guianas project, I found more taxa whose names had supposedly lost their type material when the Berlin (B) herbarium was bombed during World War II on the night of 1–2 March 1943 (Hiepko 1987: 251). Besides reading Hiepko (1987), I visited the Berlin collections of Boraginaceae in 1985 and 1987, and corresponded with Paul Hiepko and more recently with the head of the herbarium and checked the Berlin Virtual Herbarium database online without being able to substantiate the current presence at B of the types of the names lectotypified below. Typification problems in *Cordia* L. (Linnaeus 1753 : 190), *Myriopus* Small (1933: 1131), *Tournefortia* L. (Linnaeus 1753: 140), and *Varronia* P. Browne (1756: 172) are the subject of this paper.

In the citation of types, the word photo refers to a print deposited in herbaria and the word scan refers to a picture posted online, available directly through herbarium sites, or through sites like JSTOR or Europeana.

As far as I could find out, the type material of the names in the Cordiaceae and the Heliotropiaceae treated below have been destroyed at B during World War II. Lectotypes need to be designated according to Art. 9.2. of the International Code of Nomenclature for algae, fungi and plants (McNeill et al. 2012). Six such lectotypes are designated below. All the duplicates conform well to the descriptions and to the photographs of the lost specimens that are held at F and were taken by MacBride. Where the name typified is not the currently accepted name for the species, the accepted name is given beneath the type designation.

## Typifications in the Cordiaceae

### 1. Cordia discolor

Taxon classificationPlantaeBoraginalesCordiaceae

Cham., Linnaea 4: 482. 1829.

Varronia discolor (Cham.) Borhidi, Acta Bot. Hung. 34(3–4): 388. 1988.

#### Type.

Brazil. 1814, *F. Sellow s.n.* (B† [F0BN000966, photo!]; lectotype, **here designated**: LE [herb. Chamisso]; isolectotypes: HAL n.v. [HAL0098680, scan!], K! [K000583320, labelled “Ex reliquiis Sellowianis”, scan!], P! [P00634049, scan!]).

#### Accepted name.

***Varronia polycephala*** Lam.

#### Notes.

The name was given by Chamisso in 1829 to a northern South American shrub now recognized as a synonym of *Varronia polycephala* Lam. The material of *Cordia discolor* used by Chamisso and conserved at B was destroyed during World War II. I have seen the scans of the duplicates cited below as lectotype and isolectotype(s). Chamisso’s herbarium is in the collection of the Leningrad Komarov Institute (LE) in St. Petersburg. Correspondence with Irina Illarionova and Vladimir Dorofeyev (LE) resulted in the photographs of three Sellow specimens for *Cordia discolor* in the LE collections. One has “Hortus Botanicus Imperialis Petri Magni.” printed on top of the sheet and has a label on the bottom that read “Herb. Reg. Berolinense/Cordia discolor Cham. et Schl./Brasilia. Sellow legit”. It seems to be part of the general collections of the LE herbarium, but not from Chamisso’s herbarium and the identification is not from Chamisso’s hand. Another one has 2 labels; one on the bottom left reads “Martii Herbar. Florac. Brasil N° 125” and on the bottom right “Herb. Reg. Berolinense. 1845/Cordia discolor Cham. et Schl./Brasilia. Sellow legit.”. It was sent to LE after Chamisso’s deathThe third one has one label in the bottom left corner that reads “*Cordia discolor* N.” on top, “Sellow” on the bottom left, and on the bottom right “Bras. tropica” and below “Hb Cham”. It has been seen and annotated by Chamisso. It is the best choice for a lectotype.

A fragment at F labelled *Sellow 47* (GH n.v., fragment [GH00057561, scan!]) might be additional isolectotypes. In the Harvard University Herbaria Index of Specimens, F. Sellow 47 is listed as “Collector F. Sellow” and “Station 47”; the label reads “F. Sellow 472”, number that corresponds to the type of *Stylogyne pauciflora* Mez (Primulaceae). It is likely that in most cases the numbers accompanying Sellow’s collections are not collection numbers.

### 2. Cordia multispicata

Taxon classificationPlantaeBoraginalesCordiaceae

Cham. Linnaea 4: 490. 1829.

Lithocardium multispicatum (Cham.) Kuntze, Rev. Gen. 2: 977. 1891.

#### Type.

Brazil. “Eastern Brazil”, *F. Sellow s.n.* [46?] (B† [F0BN000987, photo!]; lectotype **here designated**: US! [US00110697, scan!]; isolectotypes G! [G00177047, scan!], HAL n.v. [HAL0098661, scan!]).

#### Accepted name.

***Varronia multispicata*** (Cham.) Borhidi, Acta Bot. Hung. 34(3–4): 392. 1988.

#### Notes.

The type material at B was destroyed during World War II. I am selecting as the lectotype the duplicate preserved at US that I have studied. I have also seen the duplicate at G, but have not carefully studied it.

Correspondence with Irina Illarionova and Vladimir Dorofeyev (LE) resulted in no specimen from the Chamisso herbarium collected by Sellow for *Cordia multispicata* or *Varronia multispicata* in the LE collections.

The collection *F. Sellow 46* has been cited for other groups of plants like ferns, *Asplenium sellowianun* C. Presl ex Hieron. (Meza Torres 2011: 125), Orchidaceae, *Pleurothallis sonderana* H. G. Reichenbach (Harvard University Herbaria, Index of specimens), and F. Sellow [46] Solanaceae, *Solanum convolvulus* Sendtn. (Knapp 2013: 143). It is probable that 46 is not a collection number (see above).

### 3. Cordia tobagensis

Taxon classificationPlantaeBoraginalesCordiaceae

Urb., Repert. Spec. Nov. Regni Veg. 16: 39. 1919.

Varronia tobagensis (Urb.) Borhidi Acta Bot. Hung. 34: 393. 1988.

#### Type.

Trinidad & Tobago. Tobago, 9 Sep 1912, W.E. Broadway 3072 (holotype B†; lectotype **here designated**: GH n.v. [GH00095082, scan!] fragment of the B† holotype).

#### Accepted name.

***Varronia schomburgkii*** (DC.) Borhidi

#### Notes.

The type material at B was destroyed during World War II. I am designating as the lectotype the only extant part of the original material known to me.

### 4. Cordia tobagensis Urb. var. broadwayi

Taxon classificationPlantaeBoraginalesCordiaceae

Urb., Repert. Spec. Nov. Regni Veg. 16: 40. 1919.

#### Type.

Trinidad & Tobago. Tobago, W.E. Broadway 4235 (holotype B†; lectotype **here designated**: GH n.v. [GH00095083, scan!] fragment of the B† holotype).

#### Accepted name.

***Varronia schomburgkii*** (DC.) Borhidi

#### Notes.

I am designating as the lectotype the only duplicate of the original material known to me. The lectotypes of Cordia tobagensis and var. broadwayi designated above are mounted on the same sheet at GH, but the two collections are clearly identified and have a different barcode number. At MO, the specimen *W.E. Broadway 4235* from the Grenadines is a member of the Fabaceae, *Alysicarpus* sp. (Tropicos).

### Typifications in the Heliotropiaceae

#### 5. Tournefortia paniculata

Taxon classificationPlantaeBoraginalesCordiaceae

Cham., Linnaea 4: 468. 1829.

##### Type:

Brazil. “Brazil equinoctial”, *F. Sellow s.n.* (B† [F-1053, photo!]; lectotype **here designated**: G! [G00236172, scan!]).

##### Accepted name.

***Myriopus paniculatus*** (Cham.) Feuillet, J. Bot. Res. Inst. Texas 2(1): 264. 2008.

##### Notes.

I designate as the lectotype the only duplicate known to me and that I have examined in 1982 and 1987.

#### 6. Tournefortia ulei

Taxon classificationPlantaeBoraginalesCordiaceae

Vaupel, Notizbl. Bot. Gart. Berlin–Dahlem 6: 186. 1914.

##### Type.

Bolivia. Río Madeira, Porvenir, Jan 1912, E.H.G. Ule 9711 (B† [F1063, photo!]; lectotype **here designated**: K! [K000583529, scan!]).

##### Notes.

I choose as the lectotype the specimen preserved at Kew because I was able to study it 1979 and in the 1990s.

## Supplementary Material

XML Treatment for Cordia discolor

XML Treatment for Cordia multispicata

XML Treatment for Cordia tobagensis

XML Treatment for Cordia tobagensis Urb. var. broadwayi

XML Treatment for Tournefortia paniculata

XML Treatment for Tournefortia ulei
